# Plasma proteomic changes during hypothermic and normothermic cardiopulmonary bypass in aortic surgeries

**DOI:** 10.3892/ijmm.2014.1855

**Published:** 2014-07-15

**Authors:** TEIJI ODA, AKANE YAMAGUCHI, MASAO YOKOYAMA, KOJI SHIMIZU, KOSAKU TOYOTA, TETSURO NIKAI, KEN-ICHI MATSUMOTO

**Affiliations:** 1 Division of Thoracic and Cardiovascular Surgery, Department of Surgery, Shimane University Faculty of Medicine, Shimane, Japan; 2 Department of Anesthesiology, Shimane University Faculty of Medicine, Shimane, Japan; 3 Department of Biosignaling and Radioisotope Experiment, Interdisciplinary Center for Science Research, Organization for Research, Shimane University, Shimane, Japan

**Keywords:** cardiopulmonary bypass, deep hypothermic circulatory arrest, proteomics, complement activation, biocompatibility

## Abstract

Deep hypothermic circulatory arrest (DHCA) is a protective method against brain ischemia in aortic surgery. However, the possible effects of DHCA on the plasma proteins remain to be determined. In the present study, we used novel high-throughput technology to compare the plasma proteomes during DHCA (22°C) with selective cerebral perfusion (SCP, n=7) to those during normothermic cardiopulmonary bypass (CPB, n=7). Three plasma samples per patient were obtained during CPB: T1, prior to cooling; T2, during hypothermia; T3, after rewarming for the DHCA group and three corresponding points for the normothermic group. A proteomic analysis was performed using isobaric tag for relative and absolute quantification (iTRAQ) labeling tandem mass spectrometry to assess quantitative protein changes. In total, the analysis identified 262 proteins. The bioinformatics analysis revealed a significant upregulation of complement activation at T2 in normothermic CPB, which was suppressed in DHCA. These findings were confirmed by the changes of the terminal complement complex (SC5b-9) levels. At T3, however, the level of SC5b-9 showed a greater increase in DHCA compared to normothermic CPB, while 48 proteins were significantly downregulated in DHCA. The results demonstrated that DHCA and rewarming potentially exert a significant effect on the plasma proteome in patients undergoing aortic surgery.

## Introduction

Hypothermia <32–34°C has been clinically utilized only in cardiovascular surgery with cardiopulmonary bypass (CPB) as cardiac function becomes unstable with the decrease in temperature. Profound (<20°C) to deep (<25°C) hypothermic circulatory arrest (HCA) combined with antegrade selective cerebral perfusion (SCP) is a protective strategy against brain ischemia in complex aortic surgery (1). Although there is relatively little evidence of adverse effects of profound/deep hypothermia, there is some concern that HCA may have some drawbacks, such as coagulation disorders (2,3). As a consequence, moderate HCA (25–28°C) combined with increased SCP flow rates for aortic surgery has been utilized as the mainstay of treatment (4).

The development of isobaric tag-based quantitative mass spectrometry has provided a reliable method for high-throughput analysis of proteomes (5). Proteomic changes in human plasma samples have been successfully analyzed through the use of the isobaric tag for relative and absolute quantification (iTRAQ) labeling strategy (6,7). In the present study, a comparison was made of the plasma proteomes of patients undergoing aortic surgery using deep hypothermic circulatory arrest (DHCA) and SCP (DHCA + SCP) to those undergoing aortic surgery using normothermic CPB.

## Patients and methods

### Patient selection

The present prospective cohort study received approval from the Ethics Committee of Shimane University Faculty of Medicine and was conducted at Shimane University Hospital. The patients provided written informed consent. Patients with annulo-aortic ectasia, and ascending or aortic arch aneurysms were included in this study.

### Anesthesia

The same anesthesia and CPB protocols were used for all patients: anesthesia was induced and maintained with fentanyl (15–20 μg/kg, administered intravenously prior to initiation of CPB) and propofol (3–6 mg/kg/h). Catecholamines, vasodilators and antiarrhythmic drugs were administered in a similar manner in the two groups of patients.

### CPB

The cardiopulmonary circuit was the same for the two groups, consisting of a hollow-fiber polymer-coated membrane oxygenator (QUADROX-I, HMO70000; Maquet, Rastatt, Germany), heparin-coated venous reservoir (HVR-4WF; Mera, Tokyo, Japan) and polymer-coating cardiotomy reservoir (only used in hypothermic CPB; BO-HC2821; Maquet), centrifugal pump (ROTAFLOW, BO-RF32; Maquet), arterial filter (40 μm Pall arterial line filter, AL6; Pall, Port Washington, NY, USA) and heparin-coated CPB circuit (Mera or JMS, Tokyo, Japan). The pump was primed with bicarbonated Ringer’s solution, mannitol and methylprednisolone (500 mg). After systemic anticoagulation with 3 mg/kg heparin, CPB was instituted at a flow rate of 2.0–2.8 l/min/M^2^. Blood gases, electrolytes, glucose and the hemoglobin (Hb) concentration were continuously monitored by CDI 500 (Terumo, Tokyo, Japan) and blood gas analysis was performed every 30 min (ABL800 FLEX Blood Gas Analyzer; Radiometer, Copenhagen, Denmark). Each parameter was maintained as follows: PaO_2_: 300–500 mmHg, PaCO_2_: 35–45 mmHg (α-stat management), blood glucose: 100–300 mg/dl, Hb: 7–9 g/dl in the two groups. Rectal and urinary bladder temperatures were monitored. Dilutional ultrafiltration was continuously performed during CPB for the patients in the two groups to correct hyperkalemia and fluid imbalance (Blood concentrator PLUS, BC-140 plus; Maquet). In DHCA + SCP, the body was cooled to 22°C with topical head cooling. After cardioplegic cardiac arrest and initiation of HCA, SCP was performed following cannulation into the three arch vessels and maintained by monitoring pressures (both bilateral radial artery and SCP cannula tip) and using an INVOS Oximeter (INVOS 5100C; Somanetics, Troy, MI, USA). Rewarming was initiated following completion of open distal anastomosis and initiation of distal perfusion.

### Blood sampling

Three blood samples were collected from an arterial line into ethylenediaminetetraacetic acid (EDTA) tubes as follows: T1 (before cooling, 5 min after pump initiation), T2 (before aortic declamping, 30–160 min after pump initiation in normothermic CPB or during deep hypothermia, 30–124 min after pump initiation in DHCA + SCP), T3 (just before termination of CPB, 90–193 min after pump initiation in normothermic CPB or 121–314 min after pump initiation in DHCA + SCP). Blood samples were centrifuged at 1,400 × g for 5 min, and the plasma layers were stored at −80°C.

### Immunodepletion of abundant proteins

The two most abundant plasma proteins, albumin and immunoglobulin (Ig) G, were removed using an immunodepletion column (Albumin & IgG Depletion SpinTrap; GE Healthcare, Buckinghamshire, UK) according to the manufacturer’s instructions and as previously described (7).

### iTRAQ labeling and strong cation exchange (SCX) chromatography

Samples were prepared according to the manual published by AB Sciex (Foster city, CA, USA) and as described previously (7). In brief, equal amounts of immunodepleted T1, T2, and T3 samples from each patient were denaturated and reduced, the cysteines were alkylated, and then digested with trypsin (AB Sciex). Each digest was labeled with a different iTRAQ tag using the iTRAQ reagent 4-plex kit (AB Sciex). iTRAQ label 114 was used for the T1 sample, and iTRAQ labels 115, 116 or 117 were randomly selected for the T2 and T3 samples, after which the three samples of each patient were combined. The combined samples were then fractionated into six fractions by SCX chromatography according to the manufacturer’s instructions (AB Sciex) and each fraction was desalted according to the manufacturer’s instructions (Waters, Milford, MA, USA).

### NanoLC and MALDI-TOF/TOF MS/MS analysis

One fraction from the SCX chromatography (see above) was fractionated to 171 spots using a DiNa nanoLC system (KYA Technologies, Tokyo, Japan) and collected onto an Opti-TOF LC/MALDI 384 target plate (AB Sciex) according to the manufacturer’s instructions and as previously described (7). Spotted peptide samples were analyzed by a 5800 MALDI-TOF/TOF MS/MS Analyzer with TOF/TOF Series software (version 4.0; AB Sciex). MS/MS data were analyzed using ProteinPilot™ software (version 3.0) and the Paragon™ protein database (AB Sciex). Quantitative changes of proteins at T2 or T3 were calculated using the iTRAQ ratios T2:T1 or T3:T1, respectively.

### iTRAQ data analysis and bioinformatic analysis

Proteins identified as showing expression changes were tested for conformity to the following conditions: i) a false discovery rate (FDR) <5% (FDR was estimated by ‘decoy database searching’ using the ProteinPilot Software); and ii) protein confidence >99% (‘unused ProtScore’ >2). Unused ProtScore is defined as −log (1-% confidence/100). Proteins fulfilling these criteria were considered to have ‘statistical significance’ (7,8). PANTHER software (version 8.1, http://www.pantherdb.org) was used to test for statistical overrepresentation of Gene Ontology (GO, http://www.geneontology.org) terms as described in detail elsewhere (9,10). If the number of identified genes in a GO term was significantly larger than that in the whole genome classified by the same GO term, i.e., the number of observed genes in a GO term is significantly larger than the number of expected genes in the same GO term by the binomial test, the GO term was described here as ‘overrepresented’ with statistical significance after a Bonferroni correction for multiple testing. The annotations of identified proteins were obtained from the Uniprot database (http://www.uniprot.org/).

### Western blot analysis and enzyme-linked immunosorbent assay (ELISA)

Western blot analyses were performed as described previously (7). In brief, plasma samples were separated by sodium dodecyl sulfate-polyacrylamide gel electrophoresis (SDS-PAGE), and immunoblotted using rabbit monoclonal carbonic anhydrase 1 (CA1) antibody (Abcam, Tokyo, Japan) and anti-rabbit IR dye 680-conjugated IgG (LI-COR, Lincoln, NE, USA). Protein bands were visualized using an Odyssey (LI-COR) infrared imaging system and their intensities measured for densitometric analyses of CA1. Plasma levels of the complement proteins C5a and SC5b-9 were determined using a commercially available solid-phase sandwich ELISA kit (Quidel Corp., San Diego, CA, USA) following the manufacturer’s recommendations.

### Statistical analysis

Continuous variables, such as blood gas analysis and hemodynamic variables were expressed as means ± standard deviation (SD). The variables were tested for statistically significant differences between patient groups using the Student’s t-test. Categorical variables were compared using Fisher’s exact test. For analysis of iTRAQ ratios, i.e. T2:T1 or T3:T1 ratios for each significantly identified protein, P-values were calculated by the one sample t-test of averaged protein ratio against 1 to assess the validity of the protein expression change (11). To assess ELISA and western blot analysis data, statistical comparisons were performed by two-way analysis of variance (ANOVA) (general linear model) with repeated measures followed by a post-hoc Bonferroni test to detect individual differences. The relationships between complement activation (SC5b-9) and the iTRAQ ratios of each protein including CA1 values (western blot analysis) were calculated by single and stepwise multiple regression analyses (forward selection method) (StatFlex version 6; Artech, Osaka, Japan). P<0.05 was considered to indicate statistical significance.

## Results

### Patient characteristics and CPB data

Baseline characteristics were similar in both groups except for minimal core temperature and CPB time (Table I). There were several small but significant differences in blood gas analysis, Hb and lactate levels in both groups (Table II). Pump flow rate, perfusion pressure, and core temperatures were similar between the two groups at T1 and T3. At T2, there was a significant difference in core temperature between the two groups: 23.0±1.2°C vs. 36.0±1.0°C (P<0.0001). The timing of T1 and T2 sampling from the initiation of CPB did not differ between the two groups (P=1.000 for T1, P=0.250 for T2); however, sampling at T3 was performed significantly later in the DHCA + SCP patient group than in the normothermic CPB group (Table I). The HCA time of the lower body averaged 59±41 min in DHCA + SCP patients.

### iTRAQ data analysis and bioinformatics

Mass spectrometry identified 322 proteins, of which 262 were found to be statistically significant, i.e., the proteins fulfilled both the FDR <5% and protein confidence >99% criteria. Of the 262 proteins, those that increased or decreased (>1.2-fold or <0.833-fold) in at least four of the seven samples in each treatment group were identified to obtain an adequate sample size and statistical power (5,12), subsequently analyzed by PANTHER software. The statistical overrepresentation test identified six GO categories (biological processes) with five proteins significantly increased in the normothermic CPB group at T2 (Tables III and V). By contrast, only one GO category showed significant overrepresentation in the DHCA + SCP group at T2 (Table III). At T3 in the latter treatment group, 11 GO categories were significantly overrepresented with 48 proteins significantly decreased, whereas only one GO category was significantly overrepresented in the normothermic CPB group (Tables IV and VI). In the normothermic CPB group, the iTRAQ ratio of CA1 increased significantly to 1.50±0.23 at T2 and to 1.58±0.30 at T3 in comparison to T1 (P=0.0241 at T2, and P=0.0299 at T3, Table V). The ratio also increased in the DHCA + SCP group to 1.52±0.45 at T2 and 2.65±1.65 at T3, although without statistical significance due to missing data. This increase in the CA1 ratio at T3 is a noteworthy finding as all other proteins decreased significantly in the DHCA + SCP patients, with the exception of HBA1 and HBB (Table VI).

### Western blot analysis and ELISA

**Figure 1:**
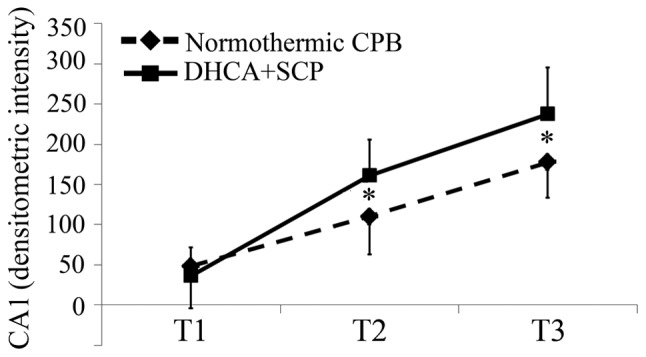
Results of the western blot analysis of carbonic anhydrase 1 (CA1) levels during cardiopulmonary bypass (CPB). CA1 levels increased significantly with time during CPB (^*^P<0.0001) in the deep hypothermic circulatory arrest (DHCA) and selective cerebral perfusion (SCP) (DHCA + SCP) and normothermic CPB patients with no significant differences between the two groups. ^*^Significant differences (P<0.0001) between sampling times in the two groups. T1, 5 min after initiation of CPB; T2, during deep hypothermia or before declamping; T3, just before termination of CPB.

**Figure 2:**
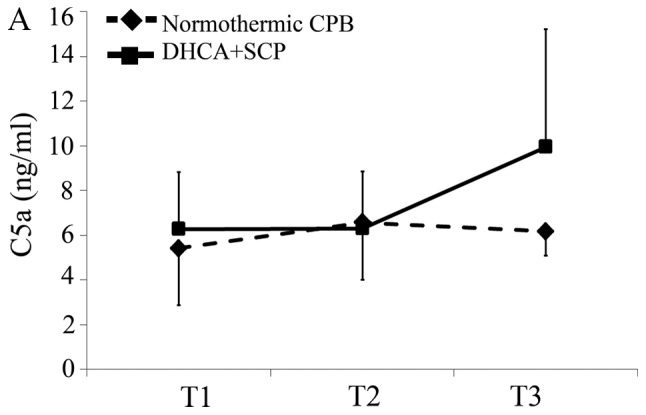

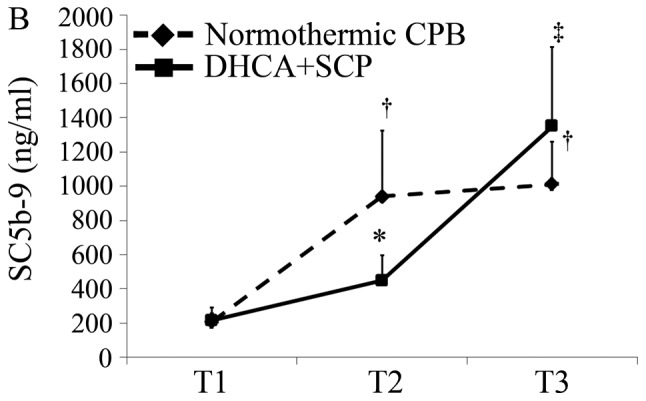
Enzyme-linked immunosorbent assay (ELISA) analyses of complement levels during cardiopulmonary bypass (CPB). (A) Levels of C5a increased non-significantly at T3 in deep hypothermic circulatory arrest (DHCA) and selective cerebral perfusion (SCP) (DHCA + SCP) patients. (B) Levels of SC5b-9 increased rapidly and significantly at T2 and, thereafter, were relatively unchanged at T3 in normothermic CPB patients. Conversely, SC5b-9 increased slightly at T2, but then increased rapidly and significantly at T3 in DHCA + SCP patients. There was a significant difference (^*^P=0.008) between the two patient groups at T2; additionally, the normothermic CPB showed significant differences (^†^P<0.0001) between T2 and T1, and between T3 and T1; DHCA + SCP patients showed significant differences (^‡^P<0.0001) between T3 and T2, and between T3 and T1. Sampling times are identical to those in Fig. 1.

To validate the iTRAQ ratios and complement activation identified by the PANTHER analysis, the protein CA1 was analyzed by western blot analysis and the complement proteins C5a and SC5b-9 were measured by ELISA. In both patient groups, the levels of CA1 continuously and significantly increased during CPB (P<0.0001), although the differences between the groups were not significant (Fig. 1). The level of complement C5a increased, albeit non-significantly, at T3 in the DHCA + SCP group compared to the normothermic group (Fig 2A). The level of SC5b-9 increased significantly at T2 in the normothermic group, and the differences between T1 and T2 and between T1 and T3 were both highly significant (P<0.0001, Fig 2B). In the DHCA + SCP group, the level of SC5b-9 increased slightly from T1 to T2, and then increased rapidly and significantly from T2 to T3. There were significant differences in SC5b-9 values between T3 and T1 and between T3 and T2 in the DHCA + SCP group (P<0.0001), and between both groups at T2, respectively (P=0.008). Since the innate immunity response is regulated by complex protein-protein interactions (13), it is difficult to clarify the causative relationship between the SC5b-9 level and iTRAQ ratios of identified proteins. Therefore, single regression analyses were performed to search for the relationships between the SC5–9 values and the iTRAQ ratios of the 42 proteins identified in all samples including the CA1 value (western blot analysis). Seven proteins (CP, HPX, VTN, F2, A1BG, GC, and CA1) showed significant correlations with SC5b-9 levels. A stepwise multiple regression analysis identified a significant coefficient (R=0.486) and ceruloplasmin as an independent variable (standardized β=−0.468, P=0.012).

## Discussion

Exposure of blood components to a CPB circuit activates various blood cell types, endothelial cells and proteins. This activation can improperly stimulate biological processes that may contribute to the development of postoperative complications resulting from the dysfunction of blood cells, coagulation, fibrinolysis, and the kinin-kallikrein and immune systems (14–16). Various modifications to the inner surface of the CPB circuit, including heparin- and polymer-coating, reduce adverse effects on complement activation, blood coagulation, proinflammatory cytokines and improve clinical outcomes (17–20). The C5 complement inhibitor pexelizumab and a complement factor 1 esterase inhibitor have been tested clinically (21) and in an animal study (22) for their abilities to reduce complement activation during CPB. Although a recent proteomic study demonstrated the possibility of identifying predictive plasma biomarkers in patients undergoing CPB (23), this approach has not been used to investigate the effect of hypothermia/rewarming on the biocompatibility of a CPB circuit.

The proteins that were identified as showing significant protein confidence in this experiment were all included in the list of proteins reported previously for the human plasma proteome (24). Our analysis using iTRAQ labeling mass spectrometry demonstrated that several GO categories related to the complement system and proteolysis were significantly overrepresented at T2 in normothermic CPB in spite of using a heparin-coated CPB circuit and polymer-coated oxygenator. Of note, these changes were not observed at T2 in DHCA + SCP patients. To confirm these findings, the levels of plasma C5a and SC5b-9 were measured by ELISA, which showed a significantly larger increase in SC5b-9 at T2 in patients undergoing normothermic CPB compared to DHCA + SCP. This reduction in the complement activation level in the DHCA + SCP group is similar to the findings from investigations of a randomized CPB study or therapeutic hypothermia for encephalopathy, which showed that hypothermia can suppress the immune response (25–27). In this context, our previous proteomic analysis of rat liver samples after a short duration (3 h) of deep hypothermia (23°C) demonstrated that 264 proteins changed significantly with 163 showing a decrease (28). Consequently, the deep hypothermia (22°C) and circulatory arrest of the lower body that occurs in DHCA + SCP might further decrease complement components (activators and regulators) that are mainly produced in the liver. Any such decrease may have a significant effect on the activity of the complement system at T2 in the DHCA + SCP patients.

Complement activation in the DHCA +SCP group was indicated by the rapid increase in the level of SC5b-9 from T2 to T3. To the best of our knowledge, this is the first study to demonstrate that CPB-induced complement activation can be initially suppressed at T2 and later upregulated at T3 in the DHCA + SCP group. The latter response may in part have occurred because complement activation can develop after rewarming. This explanation is supported by a study on mild hypothermia after cardiac arrest which demonstrated that complement activation decreased after the induction of hypothermia (32–34°C), followed by an increase during rewarming (27). Another possible cause of complement activation at T3 may be reperfusion injury of the lower part of the body that developed with the lactate production at T3 (Table II), which may indicate that the metabolism of lower body had been conducted anaerobically with the production of oxygen debt during 59±41 min of DHCA in the DHCA + SCP group (29). Moreover, rewarming has been shown to aggravate cold-induced (4°C) oxidative hepatocyte injury caused by chelatable iron via Fenton chemistry and, thereby, to accelerate complement activation (30). On the other hand, both reperfusion injury and the impact of cooling/rewarming on liver metabolism may underlie the downregulation of some of the identified proteins at T3. A number of proteins were found to be downregulated at T3 in the DHCA + SCP patients (Tables IV and VI). This downregulation seems inconsistent with the complement activation identified by the ELISA analysis. Possibly, this inconsistency might be explained by the tight control of complement activation by its regulators, suggesting that the downregulation of regulators such as vitronectin (VTN) or C1-inhibitor (SERPING1) contribute to complement activation (Table VI) (29). CA1 had a significant positive correlation with SC5b-9 levels by a single regression analysis. It has been demonstrated that CA1 causes kallikrein-kinin system activation (31). There is evidence of an interaction among the complement, coagulation and kinin systems (29,32) in which kallikrein is an initiator of the complement cascade by digestion of factor XIIa into factor XIIf (33) and possibly through the cleavage of C3 or C5 into active forms (32). By contrast, ceruloplasmin, which had a significant negative correlation with the SC5b-9 level by stepwise multiple regression analysis, was downregulated at T3 in the DHCA + SCP. This protein has ferroxidase activity to oxidize Fe^2+^ to Fe^3+^, contributing to the antioxidant capacity because of the reduction of oxidation capacity via the Fenton reaction (34). Ceruloplasmin is also reported to be an endogenous inhibitor of myeloperoxidase (35), but is downregulated by reactive oxygen species (34). Taken together, CA1, an upregulated protein at T3, may be a positive regulator of the complement system via kallikrein-kinin system activation. By contrast, ceruloplasmin, a downregulated protein at T3, may be a major negative regulator of the complement system via its antioxidant capacity. Thus, both the increased complement activator and several decreased complement regulators could result in the rapid rise of the SC5b-9 level at T3 in the DHCA + SCP patients. During normothermic CPB, the levels of C5a and SC5b-9 did not change significantly from T2 to T3 after an initial rise at T2, a finding consistent with the relatively unchanged iTRAQ ratio and with reports from previous studies (14,16).

Although we used state-of-the-art iTRAQ labeling technology mass spectroscopy, a considerable number of proteins expressed at low levels may not have been detected because of the large dynamic range in plasma proteins (24). Nevertheless, this technology may become a useful analytical tool in the field of research of biocompatibility of CPB in the future. The comparisons here involved two groups in which the two sets of patients underwent similar thoracic aortic surgery, i.e., DHCA + SCP and normothermic CPB. However, significant differences between the two groups occurred in the CPB time, which in turn affected T3 sampling time, implementation of circulatory arrest and SCP (hypothermic ischemia of lower body), and levels of PaCO_2_ and Hb at T2. These differences may affect plasma proteomic profiles. To elucidate the effects of hypothermia (and rewarming) *per se* on the biocompatibility of CPB, it may be necessary to perform a study using an animal model in the future.

In conclusion, results of the comparative iTRAQ proteomic analysis demonstrated that CPB-induced complement activation (and proteolysis) was initially suppressed during DHCA and later upregulated at the end of CPB after rewarming. Single and multiple regression analyses showed that this complement activation/suppression was associated with changes in expression of ceruloplasmin and six other proteins including CA1.

## Figures and Tables

**Table I:** Baseline characteristics, type of surgery and intraoperative variables.

Variables	Normothermic CPB (n=7)	DHCA + SCP (n=7)	P-value
Age	66.0±8.8	69.9±7.0	0.3841
Gender (male)	3	4	1.0000
β-blocker	1	4	0.2657
ACEIs/ARBs	2	6	0.1026
Calcium channel blockers	3	5	0.5921
Oral antihypoglycemic agent	1	0	1.0000
Statins	4	2	0.5921
Minimal core temperature (°C)	34.6±0.9	21.8±1.1	<0.0001
Aortic cross-clamping time (min)	107.0±31.7	117.7±68.6	0.7245
CPB time (min)	142.9±34.2	218.7±75.7	0.0457

ACEI, angiotension converting enzyme inhibitor; ARB, angiotensin II receptor blocker; CPB, cardiopulmonary bypass; DHCA + SCP, deep hypothermic circulatory arrest with selective cerebral perfusion.

**Table II:** Arterial blood gas analysis at three sampling points during CPB for aortic surgery.

Treatment methods	Sampling time	pH	PaO_2_ (mmHg)	PaCO_2_ (mmHg)	B.E.	Glucose (mg/dl)	Lactate (mmol/l)	Hb (g/dl)
	T1	7.38±0.05	387±49	41.5±4.5	−1.1±2.0	99±20	10.0±4.7	7.4±0.9
Normothermic CPB	T2	7.35±0.03	399±33	45.0±2.0^a^	−1.1±2.4	125±18	10.0±5.9	8.7±1.3^b^
	T3	7.37±0.04	373±35^c^	43.0±1.8	−0.6±1.6^d^	124±23	10.7±7.3^e^	8.3±1.4
	T1	7.38±0.03	390±38	41.4±2.6	−0.8±1.9	114±21	12.0±4.9	7.1±0.6
DHCA + SCP	T2	7.32±0.04	424±43	49.7±3.9^a^	−0.9±3.2	96±33	15.1±5.9	6.7±0.6^b^
	T3	7.35±0.03	321±28^c^	43.0±3.6	−2.3±0.8^d^	140±47	24.9±12.9^e^	7.6±0.6

Statistically significant differences between the two groups were:

a P=0.0157,

b P=0.0058,

c P=0.0102,

d P=0.0297,

e P=0.0270.

B.E., base excess; CPB, cardiopulmonary bypass; DHCA, deep hypothermic circulatory arrest; Hb, hemoglobin concentration; SCP, selective cerebral perfusion; T1, 5 min after initiation of CPB; T2, during deep hypothermia or declamping; T3, just before termination of CPB.

**Table III:** GO categories showing significant overrepresentation for plasma proteins at T2.

		*Homo sapiens*			Plasma sample	
			
GO term	Description	No. of genes^a^	No. of observed genes^b^	No. of expected genes^c^	P-value	Genes
A, Normothermic CPB						

0006956	Complement activation	99	6	0.09	6.46E-08	**C8B C1S C1R C6 C4B C5**
0006508	Proteolysis	1,131	10	1.07	3.29E-06	**C8B SERPING1 ITIH3 C1S C1R C6 C4B C5 SERPINA4 AGT**
0006955	Immune response	725	7	0.69	4.82E-04	**C8B AZGP1 C1S C1R C6 C4B C5**
0007596	Blood coagulation	271	4	0.26	1.90E-02	**C8B C1S C1R C6**
0009605	Response to external stimulus	271	4	0.26	1.90E-02	**C8B C1S C1R C6**
0019538	Protein metabolic process	3,178	10	3.02	4.02E-02	**C8B SERPING1 ITIH3 C1S C1R C6 C4B C5 SERPINA4 AGT**

B, DHCA + SCP						

0006869	Lipid transport	220	3	0.12	3.52E-02	APOD APOC3 APOA2

The test for statistical overrepresentation of each Gene Ontology (GO) term (biological process) was perfomed using PANTHER software (version 8.1). Upregulated genes are shown in bold.

a The total number of genes in the whole genomes (*Homo sapiens*, n=20,000) classified by the GO term;

b the number of genes from the input list [n=20 (mapped genes, 19; unmapped gene, 1) in the normothermic cardiopulmonary bypass (CPB) group, n=12 (mapped genes, 11; unmapped gene, 1)’ in the deep hypothermic circulatory arrest (DHCA) with selective cerebral perfusion (SCP) group (DHCA + SCP)] classified by the GO term;

c the number of genes that would be detected in the input list for a particular GO category on the basis of the reference list (*Homo sapiens*).

T2, during deep hypothermia or before declamping.

**Table IV:** GO categories showing significant overrepresentation for plasma proteins at T3.

		*Homo sapiens*			Plasma sample	
			
GO term	Description	No. of genes^a^	No. of observed genes^b^	No. of expected genes^c^	P-value	Genes
A, Normothermic CPB						

0008015	Blood circulation	192	3	0.11	2.36E-02	HP **HBA1 HBB**

B, DHCA + SCP						

0006508	Proteolysis	1131	22	2.94	3.13E-12	SERPING1 PLG SERPINC1 APOH C3 ITIH1 F2 CP A2M C4BPA C4B CFB HP SERINF2 CFH ITIH2 C5 AHSG SERPINA3 ITIH4 AMBP SERPINA1
0006956	Complement activation	99	10	0.26	1.98E-11	APOH C3 PGLYRP2 A2M C4BPA C4B CFB CFH HP C5
0007596	Blood coagulation	271	10	0.7	3.36E-07	APOH F2 CP C4BPA CFB CFH HP TF HRG AMBP
0009605	Response to external stimulus	271	10	0.7	3.36E-07	APOH F2 CP C4BPA CFB CFH HP TF HRG AMBP
0006955	Immune response	725	12	1.88	4.68E-05	A1BG APOH C3 PGLYRP2 A2M C4BPA C4B CFB CFH HP TF C5
0050896	Response to stimulus	1767	17	4.59	2.20E-04	A1BG APOH C3 PGLYRP2 F2 CP A2M C4BPA CFB C4B HP CFH TF C5 HRG AMBP
0002376	Immune system process	2480	20	6.45	2.92E-04	A1BG APOH C3 PGLYRP2 F2 CP A2M C4BPA CFB C4B HP CFH TF C5 SAA4 AHSG HRG AMBP
0019538	Protein metabolic process	3178	22	8.26	9.02E-04	SERPING1 PLG SERPINC1 APOH C3 ITIH1 F2 CP A2M C4BPA C4B CFB HP SERINF2 CFH ITIH2 C5 AHSG SERPINA3 ITIH4 AMBP SERPINA1
0006869	Lipid transport	220	7	0.57	2.89E-04	APOB APOD APOA1 APOE APOC3 APOA2 APOL1
0016337	Cell-cell adhesion	724	9	1.88	1.63E-02	APOH FGB CP C4BPA FGG CFB CFH FGA VTN
0006810	Vitamin transport	90	4	0.23	1.60E-02	TTR CP HPX RBP4

The test for statistical overrepresentation of each Gene Ontology (GO) term (biological process) was perfomed using PANTHER software (version 8.1). Upregulated genes are shown in bold.

a The total number of genes in the whole genomes (*Homo sapiens*, n=20,000) classified by the GO term;

b the number of genes from the input list [n=11 (mapped genes, 11) in the normothermic cardiopulmonary bypass (CPB) group, n=57 (mapped genes, 52; unmapped gene, 5) in the deep hypothermic circulatory arrest (DHCA) with selective cerebral perfusion (SCP) group (DHCA + SCP)] classified by the GO term;

c the number of genes that would be detected in the input list for a particular GO category on the basis of the reference list (*Homo sapiens*).

T3, just before termination of CPB.

**Table V:** Plasma proteins showing significantly changed iTRAQ ratio at T2 or T3 of normothermic CPB.

						At T2		At T3		
								
Unused ProtScore	Coverage %	Peptides (95%)	Uniprot No.	Gene	Protein	T2/T1^b^	P-value	T3/T1^b^	P-value	Biological process (GO)
33.33	96.6	39	P68871	HBB	Hb subunit β	**4.07**	0.0138	**8.70**	0.1023	Oxygen transport
19.66	73.2	30	P69905	HBA1	Hb subunit α	**3.79**	0.0216	**7.15**	0.0536	Oxygen transport
12.00	40.2	7	P00915	CA1	CA1^a^	**1.50**	0.0241	**1.58**	0.0299	Bicarbonate transport
23.29	35.0	12	P05155	SERPING1	Plasma protease C1 inhibitor	**1.29**	0.0088	1.13	0.2306	Complement activation, blood coagulation
8.01	14.3	4	P00736	C1R	Complement C1r subcomponent	**1.25**	0.0227	1.13	0.3394	Complement activation
31.70	32.4	19	P04004	VTN	VTN^a^	1.03	0.5583	*0.80*	0.0026	Innate immune response
12.01	38.1	9	P02743	APCS	Serum amyloid P-component	1.07	0.5955	*0.77*	0.0006	Acute-phase response
95.65	85.7	93	P00738	HP	Haptoglobin	0.80	0.0716	*0.62*	0.0035	Acute-phase response, defense response

Bold, increased ratio (≥1.2-fold) with statistical significance and italics, decreased ratio (≤0.83-fold) with statistical significance. Plasma samplings were performed at T1 [5 min after initiation of cardiopulmonary bypass (CPB)], T2 (before declamping) and T3 (just before termination of CPB). Unused ProtScore indicates protein confidence as expressed by the formula: ProtScore = - log (1 - % confidence/100). Coverage % indicates the percentage of matching amino acids from identified peptides. Peptides (95%) indicates the number of peptides having at least 95% confidence.

a Statistical significance of single regression analysis of relationships between isobaric tag for relative and absolute quantification (iTRAQ) ratios of proteins and SC5b-9 measured by enzyme-linked immunosorbent assay (ELISA);

b iTRAQ average ratio.

CA1, carbonic anhydrase 1; GO, Gene Ontology; Hb, hemoglobin; VTN, vitronectin.

**Table VI:** Plasma proteins showing significantly changed iTRAQ ratio at T2 or T3 in patients undergoing DHCA and SCP.

						At T2		At T3		
								
Unused ProtScore	Coverage %	Peptides (95%)	Uniprot No.	Gene	Protein	T2/T1^c^	P-value process (GO)	T3/T1^c^	P-value	Biological
19.66	73.2	30	P69905	HBA1	Hb subunit α	**4.54**	0.0011	**7.74**	0.0002	Oxygen transport
33.33	96.6	39	P68871	HBB	Hb subunit β	**4.23**	0.0009	**7.14**	0.0004	Oxygen transport
35.50	40.1	18	P19827	ITIH1	Inter-α-trypsin inhibitor heavy chain H1	1.01	0.8741	*0.83*	0.0136	Hyaluronan metabolic process
35.56	49.6	19	P01008	SERPINC1	Antithrombin-III	0.92	0.5019	*0.83*	0.0439	Blood coagulation
28.55	40.5	18	P10909	CLU	Clusterin	0.98	0.8981	*0.82*	0.0036	Innate immunity
23.29	35.0	12	P05155	SERPING1	Plasma protease C1 inhibitor	0.94	0.5123	*0.82*	0.0310	Complement pathway, blood coagulation
34.55	49.2	20	P01011	SERPINA3	α-1-antichymotrypsin	0.99	0.8905	*0.81*	0.0056	Acute-phase response
28.76	61.7	20	P02763	ORM1	α-1-acid glycoprotein 1	0.97	0.7894	*0.81*	0.0008	Acute-phase response
38.07	41.4	20	Q14624	ITIH4	Inter-α-trypsin inhibitor heavy chain H4	0.93	0.4239	*0.81*	0.0116	Acute-phase response
4.01	16.8	2	O14791	APOL1	Apolipoprotein L1	0.99	0.9045	*0.80*	0.0181	Cholesterol metabolism
45.11	33.6	27	P19823	ITIH2	Inter-α-trypsin inhibitor heavy chain H2	0.94	0.4330	*0.80*	0.0046	Hyaluronan metabolic process
20.10	45.2	12	P02760	AMBP	Protein AMBP	0.93	0.3931	*0.80*	0.0031	Negative regulation of immune response
18.81	44.2	12	P27169	PON1	Serum paraoxonase/arylesterase 1	0.87	0.0801	*0.80*	0.0306	Response to external stimulus
116.10	63.0	65	P00450	CP	CP^a^,^b^	0.97	0.7447	*0.79*	0.0012	Cellular iron ion homeostasis
100.66	74.4	92	P01009	SERPINA1	α-1-antitrypsin	0.96	0.7121	*0.78*	0.0170	Acute-phase response
29.22	35.1	16	P04196	HRG	Histidine-rich glycoprotein	0.95	0.5498	*0.78*	0.0225	Blood coagulation, chemotaxis
26.30	58.0	15	P02749	APOH β-2-glycoprotein	1	0.85	0.0556	*0.78*	0.0222	Negative regulation of blood coagulation
12.13	56.2	6	P35542	SAA4	Serum amyloid A-4 protein	*0.82*	0.0307	*0.78*	0.0176	Acute-phase response
83.67	74.9	55	P02790	HPX	Hemopexin^a^	0.96	0.6344	*0.77*	0.0013	Hb metabolic process, positive regulation of immune response
222.92	74.4	155	P01023	A2M	α-2-macroglobulin	0.93	0.4339	*0.77*	0.0027	Blood coagulation, negative regulation of complement activation
18.96	51.2	11	P02753	RBP4	Retinol-binding protein 4	0.86	0.0861	*0.77*	0.0398	Positive regulation of Ig secretion
116.37	89.9	86	P0C0L5	C4B	Complement C4-B	0.94	0.4009	*0.76*	0.0015	Complement activation
222.47	78.5	138	P01024	C3	Complement C3	0.93	0.5076	*0.76*	0.0048	Complement activation
57.40	59.5	37	P01871	IGHM	Ig μ chain C region	0.93	0.3755	*0.76*	0.0047	Immune response
39.27	61.6	23	P00734	F2	Prothrombin^a^	0.91	0.3817	*0.75*	0.0014	Acute-phase response, blood coagulation
98.95	80.0	90	P02675	FGB	Fibrinogen β chain	0.94	0.6486	*0.74*	0.0431	Blood coagulation
36.11	47.7	22	P04217	A1BG	α-1B-glycoprotein^a^	0.94	0.4631	0.74	0.0003	No description
35.34	43.1	22	P02765	AHSG	α-2-HS-glycoprotein	0.89	0.2103	0.74	0.0013	Acute-phase response, regulation of inflammatory response
84.80	51.5	51	P08603	CFH	Complement factor H	0.88	0.1187	*0.74*	0.0005	Complement activation
15.26	31.0	8	P08697	SERPINF2	α-2-antiplasmin	0.86	0.1192	*0.74*	0.0163	Acute-phase response, negative regulation of fibrinolysis
31.70	32.4	19	P04004	VTN	VTN^a^	0.94	0.4442	*0.73*	0.0014	Innate immune response, negative regulation of blood coagulation
20.01	29.6	10	P43652	AFM	Afamin	0.84	0.1878	*0.73*	0.0032	Vitamin transport
41.00	72.8	38	P01042	KNG1	Kininogen-1	0.90	0.1380	*0.72*	0.0003	Blood coagulation, inflammatory response
68.11	54.0	7	P02774	GC	Vitamin D-binding protein^a^	0.88	0.2749	*0.72*	0.0020	Vitamin D metabolic process
24.01	56.2	6	P01842	IGLC1	Ig λ chain C regions	0.87	0.2396	*0.72*	0.0041	Complement activation
63.30	74.8	52	P02679	FGG	Fibrinogen γ chain	0.92	0.5311	*0.71*	0.0275	Blood coagulation
32.48	75.2	28	P02649	APOE	Apolipoprotein E	0.87	0.0606	0.71	0.0053	Cholesterol metabolism
45.36	25.5	3	P00751	CFB	Complement factor B	0.86	0.1527	*0.71*	0.0013	Complement activation
182.88	85.7	93	P02787	TF	Serotransferrin	0.88	0.2297	*0.70*	0.0001	Cellular iron ion homeostasis
30.41	97.2	32	P01834	IGKC	Ig κ chain C region	0.89	0.3337	*0.69*	0.0002	Complement activation
87.70	43.6	24	P02647	APOA1	Apolipoprotein A-I	0.84	0.2654	*0.69*	0.0239	Cholesterol metabolism
25.19	32.3	13	P04003	C4BPA	C4b-binding protein α chain	0.91	0.4722	*0.68*	0.0005	Complement activation
47.98	54.4	24	P00747	PLG	Plasminogen	*0.82*	0.0179	*0.67*	0.0002	Fibrinolysis
273.73	45.0	144	P04114	APOB	Apolipoprotein B-100	0.86	0.0933	*0.66*	0.0032	Cholesterol metabolic process
8.00	36.4	9	P02656	APOC3	Apolipoprotein C-III	*0.82*	0.0414	*0.66*	0.0169	Lipid transport
7.45	52.4	5	P02766	TTR	Transthyretin	0.86	0.1924	*0.65*	0.0218	Retinoid metabolic process
7.31	25.5	3	Q96PD5	PGLYRP2	N-acetylmuramoyl-L-alanine amidase	*0.75*	0.0395	*0.64*	0.0001	Innate immune response
72.12	73.7	72	P01876	IGHA1	Ig α-1 chain C region	0.79	0.1176	*0.60*	0.0003	Immune response
17.02	53.4	11	P05090	APOD	Apolipoprotein D	0.74	0.0514	*0.59*	0.0002	Negative regulation of cytokine production
23.48	93.0	14	P02652	APOA2	Apolipoprotein A-II	*0.72*	0.0313	*0.58*	0.0273	Acute inflammatory response, negative regulation of cytokine secretion

Bold, increased ratio (≥1.2-fold) with statistical significance and italics, decreased ratio (≤0.833-fold) with statistical significance. Plasma samples were obtained at T1 (5 min after pump initiation), T2 (during deep hypothermia or before declamping), T3 [just before termination of cardiopulmonary bypass (CPB)]. Unused ProtScore indicates protein confidence as derived from the formula: ProtScore = −log (1-% confidence/100). Coverage % indicates the percentage of matching amino acids from identified peptides. Peptides (95%) indicate the number of peptides having at least 95% confidence.

a Statistical significance of single regression analysis of relationships between isobaric tag for relative and absolute quantification (iTRAQ) ratios of proteins and SC5b-9 measured by enzyme-linked immunosorbent assay (ELISA);

b statistical significance of stepwise multiple regression analysis of relationships between iTRAQ ratios of seven proteins identified by single regression analysis and SC5b-9;

c iTRAQ average ratio.

CP, ceruloplasmin; GO, Gene Ontology; Hb, hemoglobin; Ig, immunoglobulin; VTN, vitronectin.
